# Oral care in pregnancy

**DOI:** 10.4274/jtgga.galenos.2018.2018.0139

**Published:** 2019-11-28

**Authors:** Zeynep Yenen, Tijen Ataçağ

**Affiliations:** 1 Department of Restorative Dentistry, University of Kyrenia Faculty of Dentistry, Kyrenia, Cyprus; 2 Department of Obstetrics and Gynecology, University of Kyrenia Faculty of Medicine, Kyrenia, Cyprus

**Keywords:** Pregnancy, oral care, dental health

## Abstract

Pregnant women are susceptible to a wide range of oral health conditions that could be harmful to their own health and the future of their baby. There are many myths about the safety of dental care during pregnancy. As a result, pregnant women receive less dental care than when they are not pregnant. In our review, we tried to emphasize the importance and safety of routine dental care for pregnant women.

## Introduction

To sustain oral and dental health for a life time, effective and adequate care is essential. In women, dental care is much more important during pregnancy, breastfeeding, and menopausal periods. Pregnancy is not a disease state but instead it is a sign of being healthy. A healthy person is not expected to lose their teeth without any reason. The same rules are valid for pregnant women. If they take some simple precautions they will not have any loss of teeth or other dental problems. Nevertheless, mothers are known to face tooth decay and gingival problems during pregnancy. Due to bad oral health in pregnancy, pregnant women can experience premature delivery, low birth weight baby, pre-eclampsia, gingival tissue ulcerations, pregnancy granuloma, gingivitis, pregnancy tumors (epulis gravidarum), loose teeth, mouth dryness, and dental erosions. The changing hormone levels in pregnancy directly affect gum problems, and indirectly, tooth decay (1,2,3,4,5,6).

### Changes seen in the gums

Pregnant women undergo hormonal balance changes during pregnancy. Many tissues undergo certain changes because the placenta produces higher levels of estrogen and progesterone during pregnancy. In this period, excessive sensitivity to irritations occurs in the gingiva. In pregnancy, gingivitis or epulis gravidarum, commonly known as pregnancy tumors, can be seen very often. Pregnancy gingivitis usually starts at the second month of gestation and reaches the highest level at the eighth month, and heals spontaneously after birth. They originate from pyogenic granuloma and disappear after 1-2 months. Surgical removal is recommended if they do not vanish spontaneously. Surgical excision can be performed by conventional methods or laser. Laser treatment may give more comfortable results to the patient because pyogenic granulomas have tendency to bleed. In fact, if they do not disturb the patient and if they do not bleed excessively, there is no need for treatment during pregnancy.

Silk et al. (5) reported that gingivitis was found in 40% of pregnancies (3,4,5,6,7). However, it is argued that healthy gingiva is unaffected by pregnancy, which is only a reaction caused by increased plaque and gingivitis (8). The gum papillae may appear red, like a swollen strawberry. Fissures can occur on the edges of the gingiva and on the papillae. Bleeding, and even pain can be reported. In the ones that are in hyperplastic character gingiva is dull, light pink, and the surface is rough and dry. Sometimes all gums can be dark red in color. When the growth of the gingiva is localized to one area, then pregnancy tumors may be seen. Generally, the underlying reason is an irritant. These gingival changes are named as: Gingivitis simplex, gingivitis ulcerosa, gingivitis hypertrophicans, and pregnancy tumor. The cause of these changes has been shown to be the rising level of progesterone in the blood stream, which increases vascular permeability. Another cause of this phenomenon is thought to be the low levels of vitamin C (9). Giving vitamin C, Ca, P, and Fl is thought to be beneficial (10,11). Mothers who have attachment loss have a higher risk of giving birth to low birth weight babies (small for gestational age) when compared with mothers with healthy periodontiums (12,13,14,15,16). Periodontal diseases are related to many systemic diseases, including gestational complications.

### Changes that occur in the teeth

It is generally known that tooth decay increases during pregnancy. The teeth are painful and tooth losses can be seen. There is no scientific basis for the belief that fetal need for calcium required for intrauterine growth is obtained from the mother's teeth and that every pregnancy has tooth loss. This phenomenon can be explained by dentists as follows: nausea and vomiting are seen in 70% of pregnancies. Vomiting can affect oral hygiene negatively or may cause erosion on the maternal enamel layer. During pregnancy, a decrease in Ca concentration occurs. However, in the amount of ionized Ca, there is no difference compared with pre-pregnancy levels, although bone turnover is doubled during pregnancy. Increasing oral hygiene habits during pregnancy will help to prevent this problem.

The deterioration of oral and dental health during pregnancy depends on the following factors:

* During the first months of pregnancy some mothers may have extreme interest in some foods, especially carbohydrates, and tooth brushing can be neglected after they eat these kinds of food.

* Pregnant women bleed more readily due to the effect of pregnancy hormones (estrogen, progesterone), and may consequently avoid brushing their teeth. As a result, bacterial plaque increases. Therefore, in pregnancy, the mouth needs more care.

* Vomiting, especially during the first months of pregnancy, increases the acidic environment in the mouth. After vomiting, in the first few months, the mother may not pay enough attention to oral care. If the teeth are not brushed sufficiently, an acidic environment will form in the mouth.

* Saliva flow decreases. For these reasons, the formation of caries increases during this period.

* Mothers can neglect their own oral and dental health care while they are dealing with the health of the baby, which in turn causes a deterioration of oral health.

For these reasons, it is necessary to pay more attention to dental health care during this period (1,2,3,4,5,6,7,8,9).

### The importance of nutrition on dental health during pregnancy

Adequate intake of energy and nutrients constitutes positive health effects, and inadequate intake affects mother and baby health negatively. During this period, it is necessary for the mother to take 1200-1500 mg daily calcium for herself and her babies bones to be healthy. During pregnancy, mothers should meet their calcium requirements by taking calcium-rich foods such as milk and dairy products and green, leafy vegetables. With a good diet and adequate oral health care, there will be no different tooth problems during pregnancy. Nutrition during pregnancy is very important for general health and oral health for both the mother and the baby. Baby’s tooth development during pregnancy starts at the 5^th^ and 6^th^ weeks. The purpose of the nutrients taken in pregnancy is to balance the body’s nutritional requirements and to provide the necessary energy and nutrients for normal growth of the fetus. It has been shown that Ca and Mg values are increased in the molar teeth due to pregnancy, whereas there is no change in Zn value. It is thought that the increasing values of placental lactogen and insulin-like growth factor-1 during pregnancy is responsible for the increase in Ca (17).

### Dietry guidance during the whole pregnancy period in terms of oral and dental health;

• Fruits, vegetables, cereal, milk, dairy products, meat, fish and eggs that are rich for A, C, D vitamins, calcium and phosphorus must be taken in a balanced diet.

• Sugar should be avoided as much as possible, especially between meals.

• Dried fruit and toffees should be avoided.

• Nutrition during this period affects the health of the mother, as well as the baby, that is going to be born. The effect of vitamins A and D on enamel formation is known. There is no clear evidence that prenatal fluoride use can prevent decay (18).

### Nicotine and alcohol consumption during pregnancy

Smoking negatively affects oral health, especially the gums. Periodontal inflammation and destruction increases in patients who smoke. With the increasing number of filiform and fungiform papillae on the surface of the tongue, a so-called “smooth tongue” can be seen and therefore oral hygiene becomes difficult to control. Due to the anemic effect of smoking, wounds in the mouth may recover slowly and spontaneous bleeding can be seen (16). The oral health of the mother changes, which also affects the baby indirectly. There are around 4000 different chemicals in cigarette smoke. The acids, aldehydes, ketones, cyanide, and carbon monoxide that are among these, have direct toxic effects. Carbon monoxide, which is present in 4% of cigarette smoke, is known to prevent transport of oxygen by binding to hemoglobin in red blood cells. The hemoglobin oxygen transport capacity of smokers is reduced by 2.5% to 15%. As a result, the oxygenation of the fetus decreases, like the organs of the mother. The possibility of abortion or stillbirth increases among smokers. Also, when the baby is born alive, the birth weight is less than normal. Early or late neonatal deaths are seen much more frequently among babies of mothers who smoke (19,20).

Overuse of alcohol consumption is teratogenic in babies and can cause fetal alcohol syndrome. Epithelial growth factor receptors are responsible for dental proliferation and differentiation. Changes in these receptors due to alcohol consumption can lead to dental anomalies. Encountered findings when pregnant rats were given alcohol at certain doses, small teeth, structural deterioration of the enamel, and delayed tooth rupture were seen in young rats. It can also cause hepatic and oral pathologies in the mother and can indirectly affect the baby's condition (3,21,22,23).

### Oral care recommendations during pregnancy

The combination of personal and professional treatment during pregnancy is very important, it plays a major role in improving oral health. Zanata et al. (24) found a correlation between preventive maintenance procedures performed during pregnancy and plaque accumulation and caries prevalence (25).

* Daily oral and dental care should be continued non-stop.

* A full oral examination must be done before gestation to achieve optimal oral hygiene and gain the habit of maintaining it because there is a direct relationship between hormonal changes during pregnancy and plaque accumulation and gingival diseases. The hormone increase during pregnancy makes the mouth mucosa more sensitive to external factors, especially against bacterial plaques.

* Effective dental care should be obtained by using toothbrushes and dental floss at least twice a day.

* Gargling with mouthwashes or warm salty water must be performed. Warm salty water relaxes gums and reduces gum sensitivity.

### Treatments that can be performed during pregnancy

Many dentists think that if there is approval from the doctor of the pregnant woman they can perform uncomplicated treatments. However, most procedures to be performed in dentistry are important in the first three months and the last three months, in terms of the stresses to which the mother and the baby will be exposed. Effective dental treatment in the first trimester should be avoided. This period is a very sensitive period because it is the stage of organogenesis. Unnecessary interventions can lead to abortions. However, in cases when there is pain or if no intervention will cause more harm, the teeth must be urgently treated. Under these circumstances, tooth extraction and canal treatment can be performed. The second trimester is the most appropriate period for making many treatments, for those that if postponed until the end of pregnancy would be dangerous, such as tooth extraction, filling, and canal treatment. In the third trimester, it is not easy for the mother to take the necessary positions for the dental treatment, and may become disturbed. The baby has grown considerably in the womb and the delivery is close. It should also be remembered that if a pregnant women, in the last trimester, sits for too long in the dental chair, it may cause vena cava inferior syndrome (supine hypotensive syndrome). In this situation, turning the mother to the left side in a semi-sloping manner will help to relieve the venous circulation (1,26). Just as in the first trimester, the intervention of the dentist is not recommended except for emergency treatments.

Although some pregnant women hesitate to receive antenatal oral care, recent publications indicated that many dental treatments can be performed safely during pregnancy, such as extractions, local anesthetic, root canal treatment, scaling, and root planning (4,5).

In emergency cases such as tooth and gingival inflammation, existing infections can affect the baby’s health much more adversely than the adverse effects of dental treatment. Therefore, dental treatment must be provided according to the advice of an obstetrician.

In order to decide on the procedures to be performed, the amount of ionized radiation in a single radiograph that is taken can be reduced with lead gowns, fast films, well-calibrated instruments and collimator, which will not cause damage to the fetus. The National Radiation Protection Committee reported that the cumulative amount of radiation should not exceed 0.20 Gy, higher doses may cause microcephaly and mental retardation (27,28,29,30). Nitrous oxide used for anesthesia is known to cause abortion and congenital anomalies in pregnancy (27,31). The manufacturer’s recommendations must be taken into account in the use of local anesthetics during pregnancy for tooth extraction or any intervention. If there is no special warning, there is no inconvenience. Local anesthetics such as lidocaine and prilocain can be safely used during pregnancy in the context of the Food and Drug Administration (FDA) recommendation (1,30).

Although the mercury gases released during dental treatment are unlikely to produce teratogenic effects, they should be avoided so that the patient or workers do not inhale intensified mercury gases. In addition, it has been suggested that during the pre-pregnancy period ≥1 μg/day of mercury exposure is associated with attention-deficit/hyperactivity disorder in infants (32,33).

The ideal number of dental checks in the 1st trimester is two, and one in the second and third trimester. After a good evaluation at the first check, it should be checked whether oral hygiene is provided in the 2nd trimester and the planned treatment should be performed in this period (e.g. tooth extraction, filling).

When medication is necessary, penicillin, erythromycin, cephalosporins are safe antibiotics to use during pregnancy. However, tetracycline (coloring in teeth), vancomycin (ototoxic/nephrotoxic), streptomycin (ototoxic) have adverse effects and are inappropriate to use during pregnancy. In addition, according to ADA, ciprofloxacin, benzodiazepines, and barbiturates should be avoided absolutely. Prenatal vitamin supplement is recommended.

Pain originating from the teeth can be a reason for contractions to start, by putting the patient under stress. Therefore, it is recommended to prescribe pain relievers with consultation. Narcotic analgesics can depress the central nervous system and non-steroidal anti-inflammatory drugs can cause patent ductus arteriosus, a such their use should be avoided during pregnancy. Acetaminophen can be preferred throughout pregnancy. Pain relief medications that can be used in pregnancy, in line with FDA recommendations, are given in Table 1 (32,33).

## Conclusion

During pregnancy, oral and dental care requires special attention. Oral health is a part of general health, and it is of even greater importance during this period because it concerns both the mother and the fetus.

It should also be kept in mind that neglecting oral and dental health during pregnancy does not only cause problems such as tooth decay and tooth loss, but may also lead to problems such as premature birth, low birth weight infant, and pre-eclampsia. Pregnancy is a period in which the mother must obey certain rules in order to protect her health and her baby’s’ health. In this period, mothers can protect their oral health by taking the necessary precautions and then they can prevent dental problems that may be irreversible.

## Figures and Tables

**Table 1:**
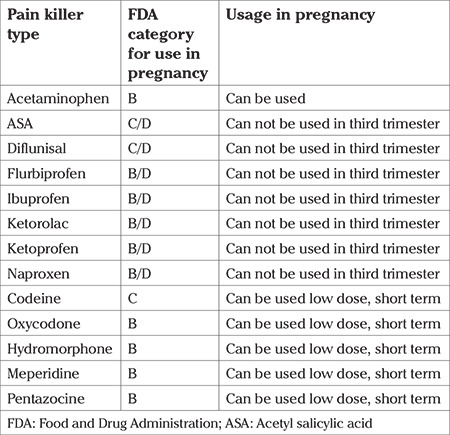
The first letter in the B/D or C/D listing indicates analgesic availability for the first two trimesters, second the third trimester
